# Grandchild Care and Its Links to Disability and Depressive Symptoms Among Urban Residents Aged 60 and Older

**DOI:** 10.3390/healthcare14152418

**Published:** 2026-08-06

**Authors:** Xinran Yu, Kailiang Shen, Patience Teaway Angeline, Ying Guan, Ying Wu

**Affiliations:** 1 School of Public Health, Southern Medical University, Guangzhou 510515, China; yxr15015880516@outlook.com (X.Y.);; 2 School of International Education, Southern Medical University, Guangzhou 510515, China

**Keywords:** grandchild care, depression, disability, China Health and Retirement Longitudinal Study

## Abstract

**Background/Objectives**: In traditional Chinese culture, grandparents often help care for grandchildren, especially in urban areas where parents face high work and life pressures. However, the physical and psychological impacts of grandchild care on older adults remain debatable. This study explored the associations between grandchild care, disability, and depressive symptoms among urban older adults, and examined whether disability mediates this link. **Methods**: Data were from the 2018 CHARLS study, including 1872 urban participants aged ≥60 years with grandchildren. Disability and depressive symptoms were assessed using the Activities of Daily Living (ADL) scale and Center for Epidemiologic Studies Depression Scale (CES-D), respectively. Group comparisons, multivariable logistic regression, and mediation analysis were performed. **Results**: Among caregivers, the prevalence of depressive symptoms was 19.2% and the prevalence of disability was 20.4%, both lower than among non-caregivers in unadjusted comparisons, though grandchild care was not independently significant in multivariable models. Female, disability, and depressive symptoms were significant risk correlates, while higher education, greater physical activity, and social participation were protective. The mediation analysis suggested that functional status partially mediated the relationship between grandchild care and depressive symptoms (proportion mediated = 33.70%). **Conclusions**: Grandchild care was associated with better physical and mental health among urban older adults in descriptive comparisons, though this association was attenuated in adjusted models. While moderate intergenerational involvement may offer social and psychological benefits, excessive caregiving burden should be prevented.

## 1. Introduction

Intergenerational care, specifically grandchild care in the context of this study, refers to the provision of childcare by grandparents, who take responsibility for raising and looking after their grandchildren [1], which happens all over the world. Influenced by traditional culture and social demands, grandchild care is prevalent among older adults in China. A study has shown that 39.07% of Chinese grandparents are involved in taking care of their grandchildren [2]. Against the backdrop of drastic socioeconomic transition, massive rural-to-urban migration among young working parents has forced grandparents to take on heavier childcare duties. In urban settings, numerous women return to full-time work right after maternity leave, prompting a growing involvement of grandparents in grandchild care to assist mothers in achieving a balance between work and family responsibilities [3]. Nevertheless, China’s childcare infrastructure remains inadequate, and elderly individuals often lack sufficient external support for grandchild care. Research has further revealed that the burden of heavy grandchild care can exert detrimental effects on older adults’ physical and psychological wellbeing [4]. Therefore, it is crucial to investigate the impact of grandchild care on the physical and mental health of elderly individuals. However, the existing research in this area remains incomplete.

To date, academic consensus on the health implications of grandchild care has not been reached. Several studies have found that grandparent caregivers report fewer depressive symptoms and higher life satisfaction, which can be attributed to enriched social roles, enhanced intergenerational reciprocity and increased daily physical activity [5,6]. On the contrary, other studies have observed neutral or even adverse health outcomes, particularly among those with intensive, long-term care tasks or multiple family care responsibilities [7,8]. These divergent findings highlight that the health effects of grandchild care are not uniform but depend on caregiving intensity, cultural context, and individual resources, underscoring the need for context-specific investigations. Three major research gaps still exist in this field. First, most previous studies focused on rural older adults or combined urban and rural samples, while urban and rural groups differ greatly in living conditions, access to public services and caregiving expectations. Urban grandparents have better access to welfare services and mainly provide low-intensity daytime childcare [9], while rural grandparents often undertake long-term care for left-behind children with limited support, suffering stronger adverse health impacts. Second, the existing literature prioritizes mental health over physical function, and rarely integrates grandchild care, disability, and depressive symptoms into a unified analytical framework. Third, although disability and depression are closely correlated in late life, the potential mediating pathway linking grandchild care, functional limitation, and mental health has not been fully clarified for urban older populations.

Accordingly, we propose a conceptual framework that considers two hypothesized pathways linking grandchild care and depressive symptoms: (1) a direct psychological pathway, in which caregiving enhances older adults’ sense of purpose, self-identity, and social integration and thereby alleviates depressive symptoms; (2) an indirect physical pathway, whereby grandchild care encourages physical activity and social engagement, preserves physical function and reduces the risk of disability; improved physical functioning further relieves depressive symptoms. These pathways are examined as cross-sectional associations rather than as causal mechanisms.

The purpose of this study is to utilize data from the 2018 China Health and Retirement Longitudinal Study (CHARLS) to gain insight into the current situation of grandchild caregiving among urban elderly individuals in China. The objectives of this research are to analyze the associations between grandchild care and disability and depression among urban elderly individuals, explore the potential mediating effects between grandchild care, disability, and depression among urban elderly individuals. The findings are expected to provide practical implications for reducing disability and depressive symptoms among elderly caregivers and improving their overall wellbeing.

## 2. Materials and Methods

### 2.1. Participants

**Figure 1:**
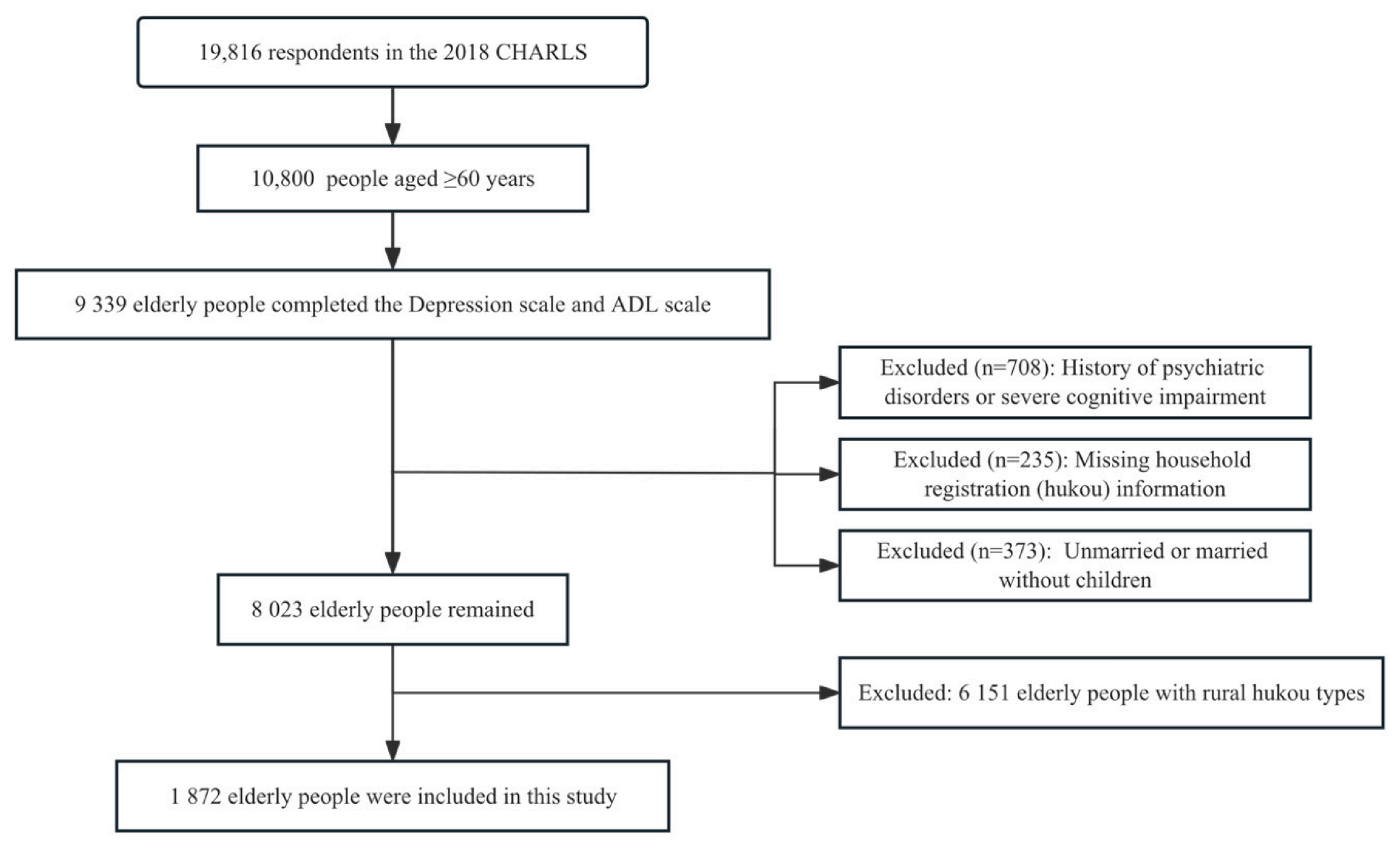
Flow diagram of participant enrolment and sample derivation.

This study used data from the 2018 wave of the China Health and Retirement Longitudinal Study (CHARLS) [10], a nationally representative survey of Chinese adults aged 45 years and older. CHARLS began with its baseline survey in 2011 and subsequently conducted national follow-up waves in 2013, 2015, 2018, and 2020. Given that data released in 2020 were potentially influenced by the COVID-19 pandemic, the 2018 wave was considered more capable of reflecting the normative relationship between grandchild care and depressive symptoms under ordinary social circumstances. A total of 1872 urban participants aged 60 years and older who had at least one grandchild were included in the analysis. Participants were included in the present analysis if they met all of the following criteria: (1) aged 60 years or older; (2) completed the relevant assessments for disability and depressive symptoms; (3) had no history of mental disorders or severe cognitive impairment; (4) had complete urban hukou (household registration) information; and (5) had at least one grandchild. Criterion (5) excludes unmarried individuals and married older adults without children, as they cannot provide grandchild care. Respondents were further excluded if they presented missing data on key study variables. The final analytic sample included 1872 urban older adults. The selection process is shown in Figure 1.

### 2.2. Variables and Instruments

We extracted demographic characteristics (age, gender, and education level), family characteristics (spouse and number of children), medical insurance, lifestyle factors (drinking, smoking, and physical activity intensity), interpersonal interactions (social activities and contact with children), Activities for Daily Living (ADL) scores, and CES-D scores from the study population. Grandchild care was coded as 1 when the respondent or their spouse had spent time caring for a grandchild during the past year and as 0 otherwise. Physical activity intensity was classified according to weekly physical activity energy expenditure, calculated as metabolic equivalent (MET) × daily activity time × number of activity days per week, and grouped into low (<600 METs/week), moderate (600–3000 METs/week), and high (>3000 METs/week). Other variables are defined in Table 1.

The physical health of the elderly was evaluated using the ADL scale [11], which consists of two parts: the Bodily Activities for Daily Living (BADL) and the Instrumental Activities of Daily Living (IADL). The BADL includes bathing, dressing, eating, toileting, grooming, and walking; the IADL includes housework, cooking, shopping, making phone calls, taking medication, and managing money. Each item was rated as no difficulty, difficulty but can be accomplished alone, difficulty and needs help, cannot be accomplished. Since caring for grandchildren requires a lot of responsibility for daily living, our study uses a more restrictive way of defined disability. A virtual variable is constructed for disability, assigning a value of 0 if there is no difficulty in completing any of the 12 activities, and conversely assigning a value of 1 to assess disability [12,13].

The mental health status of the elderly was assessed by the Centre Epidemiological Studies Depression Scale (CES-D). It consists of 10 symptomatic entries, each of which is scored on a 4-point Likert scale, with a total score ranging from 0 to 30—the higher the score, the worse the psychological condition. In this study, A CES-D score of 10 or higher defined the presence of depressive symptoms [14].

### 2.3. Statistical Analysis

First, normality and homogeneity of variance tests were conducted for continuous variables. For data following a normal distribution and meeting the homogeneity of variance assumption, descriptive statistics were presented as means ± standard deviations (SD), and independent samples T-test were used to compare group means between grandchild caregivers and non-caregivers. For data not conforming to a normal distribution, non-parametric tests (e.g., Mann–Whitney U test) were applied, and descriptive results were presented as medians with interquartile ranges (IQRs). Demographic characteristics of urban older adults were described using frequencies and percentages. The Chi-square test was used to compare the prevalence of depressive symptoms and disability between grandchild caregivers and non-caregivers. Multivariate logistic regression analyses were conducted to estimate adjusted odds ratios (ORs) and 95% confidence intervals (CIs) for correlates of depressive symptoms and disability among urban older adults.

In order to test the mediating role of disability in the relationship between grandchild care and depressive symptoms, a bootstrap analysis with 5000 resamples was performed. No covariates were included in the mediation models. This crude analysis was prespecified as an exploratory description of cross-sectional associations rather than as an estimate of adjusted or causal mediated effects. The indirect effect was considered statistically significant if the 95% confidence interval did not include zero. All statistical analyses were performed using IBM SPSS StatisticsV27.0, and *p*-value < 0.05 was considered statistically significant.

Depression and disability were treated as binary variables in Chi-square and logistic regression analyses to quantify their independent categorical associations with grandchild caregiving. By contrast, they were examined as continuous indicators, namely the CES-D score and ADL score, in mediation analyses to delineate the chained pathway. This dual analytical strategy was purpose-built to answer distinct but complementary research questions and generate different target effect sizes. Logistic regression reports odds ratios for binary outcomes, and mediation partitions the total exposure effect into direct and disability-mediated indirect effects using continuous indicators. Continuous scoring preserves fine-grained variation in symptoms and function lost during dichotomization.

## 3. Results

### 3.1. Sociodemographic Characteristics of Urban Older Adults

Among the 1872 elderly people with urban household registration, 869 (46.4%) provided grandchild care. Overall, 803 participants (42.9%) were older than 70 years, and men slightly outnumbered women. The educational distribution was relatively balanced; 342 participants (18.3%) had no spouse, including those who were divorced or widowed; and 757 (40.4%) had two children. Moderate-intensity physical activity was reported by 820 participants (43.8%). The proportions reporting drinking, smoking, or social participation were similar, and few participants did not have weekly contact with their children. Overall, 495 participants (26.4%) had disability and 415 (22.2%) had depressive symptoms (Table 2).

Compared with those who do not look after their grandchildren, the age of the elderly who care for their grandchildren is mainly 65–69 years old, their education is mainly junior high school and above, the proportion of the elderly who have a spouse do high-intensity physical activities, and weekly contact with their grandchildren are higher. The proportion of elderly people who took care of grandchildren was significantly lower than the proportion of elderly people who did not take care of grandchildren, both in terms of disability and depression (Table 2).

### 3.2. Comparison of Depression and Disability Scores Among Urban Older Adults

As shown in Table 3, caregivers had lower mean CES-D scores than non-caregivers (6.17 vs. 7.19; t = 3.825, *p*-value < 0.001). Caregivers also had lower mean ADL scores (0.51 vs. 0.77; t = 3.773, *p*-value < 0.001). These differences indicate that caregivers in this sample had fewer depressive symptoms and lower levels of functional limitation than non-caregivers.

### 3.3. Logistic Regression Analysis of Factors Associated with Depression and Disability Among Urban Older Adults

The results of multiple logistic regression analyses showed that gender and disability were risk factors for depression in urban elderly people, women were approximately 1.5 times more likely to have depressive symptoms than men; and the probability of depression in disability elderly people was approximately 3.0 times that of normal elderly people. Education level, intensity of physical activity, and participation in social activities were protective factors for the occurrence of depression in elderly people. Elderly people with a high school education or above had a 52% lower risk of depression than those who were illiterate; elderly people who could do medium and high physical activity intensities had about a 40% lower risk of depression than those who did low physical activity intensities; elderly people who participate in social activities have a 22% lower risk of depression. Smoking and drinking were not statistically significantly associated with depression in the adjusted model (Table 4).

Age, gender, and number of children and depression were risk factors for disability in urban elderly people. The risk of disability was 50% higher in people over 70 years of age; women’s risk of disability was about 1.7 times higher than men’s; the risk of disability in elderly people with more than three children was nearly twice as high; elderly people with depressive symptoms were about 3.0 times more likely to be disabled than normal people. Education level, intensity of physical activity, and participation in social activities were protective factors for the disability in elderly people. Elderly people with a high school education or above had a 43% lower risk of disability than those who were illiterate; elderly people who could do high physical activity intensities had about a 68% lower risk of disability than those who did low physical activity intensities; elderly people who participated in social activities had a 26% lower risk of disability. Smoking and drinking were not statistically significantly associated with disability in the adjusted model (Table 4).

### 3.4. Mediation Effect of Disability on the Relationship Between Grandchild Care and Depressive Symptoms

CES-D score and ADL score were positively correlated (r = 0.348, *p*-value < 0.001). In order to explore whether there was a mediating effect between grandchild care and depression and disability, we used a linear regression method to develop three models.

Model 1 estimated the total association between grandchild care and CES-D score, with CES-D score as the dependent variable and grandchild care as the independent variable. Grandchild care was significantly associated with CES-D score (*p*-value < 0.001).

Model 2 examined the effect of grandchild care on disability, with ADL score as the dependent variable and grandchild care as the independent variable. The results showed that grandchild care was significantly associated with ADL score (*p*-value < 0.001).

Model 3 included both grandchild care and ADL score as independent variables, with CES-D score as the dependent variable in order to test whether disability mediated the association between grandchild care and depression. After ADL score was entered into the model, grandchild care remained significantly associated with CES-D score (*p*-value = 0.007), and ADL score was also significantly associated with CES-D score (*p*-value < 0.001). Compared with Model 1, the regression coefficient for grandchild care decreased from −1.012 to −0.671 after inclusion of ADL score, suggesting that disability played a partial mediating role in the association between grandchild care and depression (Table 5).

**Figure 2:**
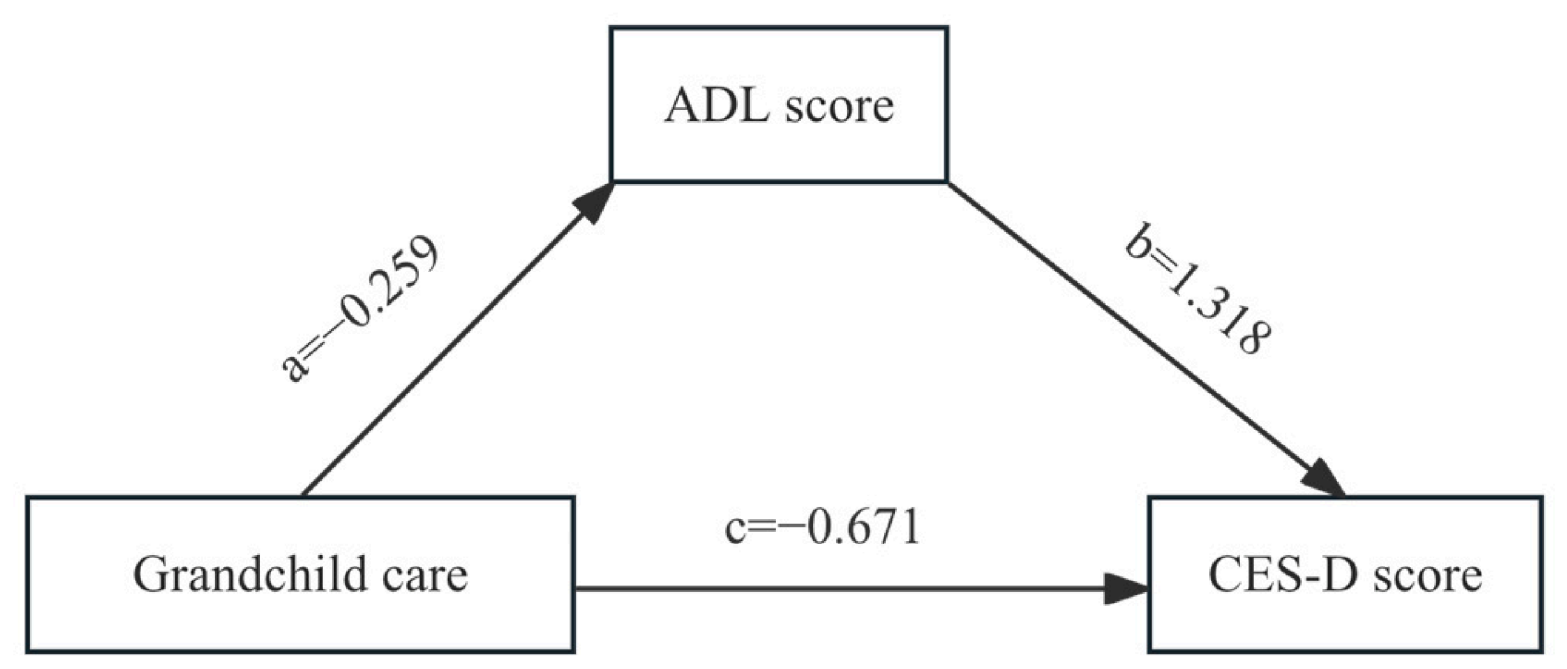
Mediation effect of ADL score on the grandchild care–CES-D score relationship.

We assumed that ADL score was a mediating factor between grandchild care and CES-D score. The bootstrap analysis estimated a total association of −1.012 between grandchild care and CES-D score and a direct association of −0.671. The estimated indirect association through ADL score was −0.342 (95% CI: −0.522 to −0.165), and the confidence interval excluded zero (Table 6 and Figure 2), which suggests that there is a partially mediated effect of ADL score between grandchild care and CES-D score (Table 6, Figure 2).

## 4. Discussion

### 4.1. Grandchild Care and the Physical and Mental Health of Urban Older Adults

In recent years, the occurrence of psychiatric disorders such as depression in elderly people in China have attracted the attention of society. Recent evidence indicates that depressive symptoms remain common among older adults, and nationwide studies continue to identify a substantial burden and multiple risk profiles [15]. The incidence of depressive symptoms in elderly people reported by Zhang [16] in 2018 was 32.2%, and the results of the analysis of CHARLS data based on 2013, 2014, and 2018 by Du [17] in 2022 showed that the incidence of depression in elderly people was 31.2%. The results of our study showed that the incidence of depression among urban elderly caring for grandchildren is 19.2%, which is lower than what has been reported in broader samples, suggesting that grandchild care may exert a protective effect against depressive symptoms. Nevertheless, this disparity could be driven by differences in measurement instruments, sample composition, and participants’ baseline health.

Meanwhile, the incidence of disability among the elderly who took care of grandchildren was 20.4%, which was lower than that of the elderly who did not take care of grandchildren in unadjusted comparisons, suggesting an association between grandchild care and better physical health outcomes. These findings are broadly consistent with the study by Wang [6], who reported that grandparenting may improve mental wellbeing partly through children’s support, and with the findings of Mou [7], who suggested that moderate grandchild caregiving may be associated with fewer depressive symptoms. In the Chinese family context, caring for grandchildren is often regarded as an important form of intergenerational support, and moderate involvement in such care may strengthen grandparents’ sense of fulfilment and self-identity. However, the health effects of grandchild care are not always uniform. Xie and Wang [18] found that high-intensity grandchild care may be associated with greater biological aging, especially among grandmothers. Therefore, the findings of this study support the view that grandchild care may be associated with better health when provided at an appropriate intensity, but excessive caregiving may become a source of strain.

### 4.2. Risk and Protective Factors Associated with Depression and Disability

In this study, multiple logistic regression showed that gender and number of children were risk factors for both disability and depression among urban older adults. Women had a significantly higher risk of disability and depressive symptoms than men, which is consistent with the findings of Zhao et al. [19]. This may be related to the fact that grandmothers usually devote more time and effort to caring for grandchildren and often undertake more household responsibilities than grandfathers, as also suggested by Xie and Wang [18], and are more likely to be depressed than grandfathers due to the fact that women’s mental health is easily affected by hormone secretion [20].

The number of children was also associated with poorer physical and mental health. One possible explanation is that as the number of children increases, older adults may face more frequent and more complicated grandchild caregiving demands across different households. This may increase both physical burden and emotional pressure, thereby raising the risk of disability and depression.

This study has shown that education level, physical activity intensity, and social activities are protective factors for older people’s physical and mental health in the adjusted models. The higher the level of education, the better the psychological condition of the elderly, indicating a significant association between education and depression, a result consistent with previous studies [21]. Education can indirectly affect depression symptoms in older adults by influencing economic level, lifestyle, and cognitive level, and can also directly affect the symptoms of depression in the elderly [22]. Higher physical activity intensity was associated with lower risks of depression and disability. This association is in line with the findings of Ge [23], who reported that moderate-to-vigorous physical activity was associated with lower odds of depressive symptoms in middle-aged and older Chinese adults. At the same time, this relationship may also reflect the fact that older adults with better physical and mental health are more able to engage in physical activity, whereas those with depression or functional limitations may reduce their activity level.

Older adults who actively participate in social activities, by interacting with their peers, can be releasing stress and reduce the lack of social presence that comes with aging. It is not only good for mental health, but also positive for physical health [24]. This study has shown that social activity participation among urban older adults has a significant negative effect on both their activities of ADL scores and CES-D score. This interpretation is consistent with the findings of Zeng [25], who emphasized the importance of late-life social participation, and with the findings of Zhang [26], who showed that lower social activity diversity was associated with worse ADL trajectories in older adults.

### 4.3. Mediating Role of Disability in the Association Between Grandchild Care and Depression

This study’s mediation analysis suggested a partial mediating effect of disability in the association between grandchild care and elderly depression, consistent with a theoretical pathway in which grandchild care is associated with lower disability, which in turn is associated with fewer depressive symptoms. Specifically, grandchild care may be linked to lower depressive symptoms through two statistically identified pathways. First, it exerts a direct psychological benefit: providing grandchild care may enhance older adults’ sense of life value, family contribution, and self-identity, thereby reducing the risk of depression. Second, it plays an indirect protective role via physical functioning. Grandchild care increases daily physical activity, improves physical health, and lowers the risk of disability, which in turn alleviates depressive symptoms.

The robust linkage between disability and depression in later life is further supported by recent longitudinal evidence. Zhang [27] identified a bidirectional dynamic relationship between ADL disability and depressive symptoms among older adults. Accordingly, this study demonstrates that physical disability serves as a crucial mediating pathway through which grandchild care shapes the mental health of older adults, highlighting the necessity of considering physical functioning when interpreting the health implications of grandparental caregiving.

### 4.4. Limitations

However, several limitations should be acknowledged. First, the cross-sectional design cannot determine temporal ordering to support causal inference, and our unadjusted exploratory mediation analysis only describes crude correlations of continuous outcomes rather than identifying causal mediated pathways. Second, grandchild care was measured as a binary variable, which fails to capture variations in care intensity, frequency, and duration. These multidimensional characteristics are critical for understanding differentiated health outcomes, and future studies should adopt more granular measurements of caregiving. Third, all information regarding disability, depressive symptoms, and lifestyle behaviors was self-reported, which may introduce recall bias and social desirability bias. Finally, the data were collected in 2018. Subsequent changes in fertility policies, childcare expectations, and labor market conditions may limit the generalizability of the findings to the current context.

## 5. Conclusions

In conclusion, this study highlights associations between grandchild care and better physical and mental health among urban older adults. Relative to non-caregivers, older adults engaged in grandchild care exhibited a lower prevalence of depressive symptoms and disability. After adjusting for covariates, the protective association between grandchild care and poor mental/physical health was weakened. Consistent with previous studies, gender and number of children were important correlates of older adults’ physical and mental health.

Education, physical activity, and social engagement emerged as protective factors in the adjusted models. Higher educational attainment correlated with better mental health, whereas greater physical activity and social participation were linked to improved overall wellbeing.

Our exploratory unadjusted mediation analysis indicated functional disability acts as an intermediate pathway linking grandchild care and depressive symptoms. Grandchild care may alleviate depressive symptoms partly by preserving physical function: the increased physical activity associated with caregiving could reduce the risk of disability and subsequently promote mental wellbeing. Nevertheless, given the cross-sectional design of this study, we cannot confirm causal pathways or directional mediation effects. Future longitudinal follow-up research is needed to validate the temporal sequence of exposures, mediators and outcomes, and construct covariate-adjusted mediation models based on standardized causal identification assumptions. Prospective longitudinal designs will facilitate rigorous formal causal mediation analysis to comprehensively clarify the association between grandparental caregiving and depressive symptoms in older adults, as well as the concrete mediating mechanisms underlying this relationship.

In summary, although grandchild care correlates with favorable health outcomes in this sample, maintaining appropriate and balanced caregiving arrangements remains vital to safeguard older caregivers’ health. From a practical perspective, this study underscores the need for nuanced, targeted interventions for urban grandparents engaged in childcare. Policies and community services should acknowledge both the potential health benefits and risks of grandchild care. Expanded public childcare resources and ongoing health support can relieve excessive care burdens, enable older adults to maintain manageable caregiving responsibilities, and help prevent disability and depressive symptoms.

## Figures and Tables

**Table 1:** Description of variables used in this study.

Variables	Variable Assignment
Grandchild care	No = 0; Yes = 1
Age	60–64 = 1; 65–69 = 2; ≥70 = 3
Gender	Male = 0; Female = 1
Education	Illiterate = 1; Below elementary school = 2; Elementary school = 3; Middle school = 4 High school and above = 5
Spouse	No = 0; Yes = 1
Number of children	1 = 1; 2 = 2; 3 = 3; ≥4 = 4
Medical insurance	No = 0; Yes = 1
Physical activity intensity	Low = 1; Medium = 2; High = 3
Drinking	No = 0; Yes = 1
Smoking	No = 0; Yes = 1
Social activities	No = 0; Yes = 1
Weekly contacts with children	No = 0; Yes = 1
Disability	No = 0; Yes = 1
Depression	No = 0; Yes = 1

**Table 2:** Characteristics of urban older adults stratified by grandchild care status.

Variables		Grandchild Care		Total	χ^2^ (*p*-Value)
		No (*n* = 1003)	Yes (*n* = 869)		
Age	60–64	187 (18.6)	338 (38.9)	525 (28.0)	243.413 (<0.001)
	65–69	220 (21.9)	324 (37.3)	544 (29.1)	
	≥70	596 (59.4)	207 (23.8)	803 (42.9)	
Gender	Male	541 (53.9)	481 (55.4)	1022 (54.6)	0.375 (0.540)
	Female	462 (46.1)	388 (44.6)	850 (45.4)	
Education	Illiterate	142 (14.2)	77 (8.9)	219 (11.7)	30.548 (<0.001)
	Below elementary school	140 (14.0)	92 (10.6)	232 (12.4)	
	Elementary school	264 (26.3)	200 (23.0)	464 (24.8)	
	Middle school	229 (22.8)	251 (28.9)	480 (25.6)	
	High school and above	228 (22.7)	249 (28.7)	477 (25.5)	
Spouse	No	248 (24.7)	94 (10.8)	342 (18.3)	60.324 (<0.001)
	Yes	755 (75.3)	775 (89.2)	1530 (81.7)	
Number of children	1	110 (11.0)	247 (28.4)	357 (19.1)	165.259 (<0.001)
	2	367 (36.6)	390 (44.9)	757 (40.4)	
	3	257 (25.6)	139 (16.0)	396 (21.2)	
	≥4	269 (26.8)	93 (10.7)	362 (19.3)	
Medical insurance	No	39 (3.9)	15 (1.7)	54 (2.9)	7.770 (0.005)
	Yes	964 (96.1)	854 (98.3)	1818 (97.1)	
Physical activity intensity	Low	149 (14.9)	94 (10.8)	243 (13.0)	23.413 (<0.001)
	Medium	471 (47.0)	349 (40.2)	820 (43.8)	
	High	383 (38.2)	426 (49.0)	809 (43.2)	
Drinking	No	505 (50.3)	400 (46.0)	905 (48.3)	3.478 (0.062)
	Yes	498 (49.7)	469 (54.0)	967 (51.7)	
Smoking	No	536 (53.4)	452 (52.0)	988 (52.8)	0.380 (0.538)
	Yes	467 (46.6)	417 (48.0)	884 (47.2)	
Social activities	No	482 (48.1)	379 (43.6)	861 (46.0)	3.699 (0.054)
	Yes	521 (51.9)	490 (56.4)	1011 (54.0)	
Weekly contacts with children	No	91 (9.1)	34 (3.9)	125 (6.7)	19.896 (<0.001)
	Yes	912 (90.9)	835 (96.1)	1747 (93.3)	
Disability	No	685 (68.3)	692 (79.2)	1377 (73.6)	30.765 (<0.001)
	Yes	318 (31.7)	177 (20.4)	495 (26.4)	
Depression	No	755 (75.3)	702 (80.8)	1457 (77.8)	8.188 (0.004)
	Yes	248 (24.7)	167 (19.2)	415 (22.2)	

**Table 3:** Depression and disability scores stratified by grandchild care status.

Variables	Grandchild Care		*t*-Value	*p*-Value
	No (*n* = 1003)	Yes (*n* = 869)		
CES-D score	7.19 ± 5.92	6.17 ± 5.46	3.825	<0.001
ADL score	0.77 ± 0.58	0.51 ± 0.14	3.773	<0.001

**Table 4:** Multivariable logistic regression analyses of depression and disability among urban older adults.

Variables		Depression		Disability	
		OR (95% CI)	*p*-Value	OR (95% CI)	*p*-Value
Grandchild care	No	1		1	
	Yes	0.916 (0.708, 1.185)	0.502	0.902 (0.701, 1.162)	0.425
Age	60–64	1		1	
	65–69	0.797 (0.579, 1.098)	0.166	0.873 (0.627, 1.215)	0.421
	≥70	0.842 (0.609, 1.164)	0.298	1.547 (1.123, 2.130)	0.008
Gender	Male	1		1	
	Female	1.475 (1.014, 2.144)	0.042	1.753 (1.214, 2.531)	0.003
Education	Illiterate	1		1	
	Below elementary school	0.760 (0.497, 1.162)	0.205	1.269 (0.834, 1.932)	0.266
	Elementary school	0.652 (0.446, 0.954)	0.027	1.045 (0.715, 1.525)	0.822
	Middle school	0.618 (0.417, 0.917)	0.017	0.721 (0.484, 1.072)	0.106
	High school and above	0.483 (0.317, 0.735)	<0.001	0.566 (0.373, 0.861)	0.008
Spouse	No	1		1	
	Yes	0.854 (0.633, 1.153)	0.303	0.951 (0.709, 1.275)	0.736
Number of children	1	1		1	
	2	1.242 (0.875, 1.762)	0.225	1.238 (0.862, 1.778)	0.248
	3	1.208 (0.803, 1.817)	0.365	1.614 (1.077, 2.421)	0.021
	≥4	1.021 (0.661, 1.576)	0.926	1.953 (1.283, 2.973)	0.002
Medical insurance	No	1		1	
	Yes	0.612 (0.331, 1.132)	0.118	0.945 (0.512, 1.745)	0.856
Physical activity intensity	Low	1		1	
	Medium	0.556 (0.398, 0.778)	<0.001	0.521 (0.377, 0.721)	<0.001
	High	0.585 (0.413, 0.828)	<0.001	0.318 (0.226, 0.447)	<0.001
Drinking	No	1		1	
	Yes	1.059 (0.803, 1.398)	0.685	0.966 (0.737, 1.265)	0.801
Smoking	No	1		1	
	Yes	1.097 (0.777, 1.548)	0.599	1.145 (0.814, 1.609)	0.437
Social activities	No	1		1	
	Yes	0.776 (0.611, 0.987)	0.038	0.739 (0.586, 0.932)	0.011
Weekly contacts with children	No	1		1	
	Yes	0.836 (0.535, 1.306)	0.431	0.663 (0.433, 1.015)	0.058
Disability	No	1		-	-
	Yes	3.033 (2.360, 3.898)	<0.001	-	-
Depression	No	-	-	1	
	Yes	-	-	3.061 (2.384, 3.930)	<0.001

CI: Confidence interval. OR: Odds ratio.

**Table 5:** Linear regression analysis of factors associated with CES-D score and ADL score among urban older adults.

	Model 1				Model 2				Model 3			
	b	SE	*t*	*p*-Value	b	SE	*t*	*p*-Value	b	SE	*t*	*p*-Value
Constant	7.185	0.183	39.817	<0.001	0.766	0.047	16.347	<0.001	6.171	0.181	34.059	<0.001
Grandchild care	−1.012	0.265	−3.825	<0.001	−0.259	0.069	−3.773	<0.001	−0.671	0.249	−2.684	0.007
ADL score	-	-	-	-	-	-	-	-	1.318	0.084	15.763	<0.001

Note. b: Unstandardized regression coefficient. SE: Standard error.

**Table 6:** Breakdown table of total effects, direct effects, and intermediary effects.

	Value	SE	95% CI	Effect Proportion
Direct effect (grandchild care → CES-D score)	−0.671	0.249	(−1.160, −0.181)	66.30%
Mediating effect (grandchild care → ADL score → CES-D score)	−0.342	0.091	(−0.522, −0.165)	33.70%
Total effect	−1.012	0.265	(−1.531, −0.493)	-

Note. SE: Standard error.

## Data Availability

The datasets used in this study are publicly available on the China Health and Retirement Longitudinal Study (CHARLS) website at https://charls.pku.edu.cn/. Data generated and analyzed during this study are available from the corresponding author upon reasonable request.
